# Recruitment of naive CD4^+^ T cells by the recombinant zoster vaccine correlates with persistent immunity

**DOI:** 10.1172/JCI172634

**Published:** 2023-12-01

**Authors:** Kerry J. Laing, Emily S. Ford, Michael J. Johnson, Myron J. Levin, David M. Koelle, Adriana Weinberg

**Affiliations:** 1 Department of Medicine, University of Washington, Seattle, Washington, USA.; 2 Vaccine and Infectious Diseases Division, Fred Hutchinson Cancer Center, Seattle, Washington, USA.; 3 Department of Pediatrics, University of Colorado School of Medicine and; 4 Department of Medicine, University of Colorado School of Medicine, University of Colorado Denver, Anschutz Medical Campus, Aurora, Colorado, USA.; 5 Department of Laboratory Medicine and Pathology and; 6 Department of Global Health, University of Washington, Seattle, Washington, USA.; 7 Translational Medicine, Benaroya Research Institute, Seattle, Washington, USA.; 8 Department of Pathology, University of Colorado School of Medicine, University of Colorado Denver, Anschutz Medical Campus, Aurora, Colorado, USA.

**Keywords:** Vaccines, Adaptive immunity, T cell receptor

## Abstract

Herpes zoster (HZ) is a substantial problem for people with decreased cell-mediated immunity, including older adults. The first vaccine approved for HZ prevention, the zoster vaccine live (ZVL), which provided limited and short-lived protection, has been supplanted by the superior recombinant zoster vaccine (RZV), which provides robust and durable protection. To understand the mechanisms underlying the differential immunologic characteristics of the 2 vaccines, we used T cell receptor β chain sequencing and peptide–MHC class II tetramer staining to analyze recombinant glycoprotein E–specific (gE-specific) CD4^+^ T cell clonotypes in RZV and ZVL recipients. Compared with ZVL, RZV expanded more gE-specific CD4^+^ clonotypes, with greater breadth and higher frequency of public clonotypes. RZV recruited a higher proportion of clonotypes from naive than from memory cells, while ZVL recruited equally from memory and naive compartments. Compared with memory-derived, naive-derived clonotypes were more likely to last 5 or more years after immunization. Moreover, the frequency of tetramer^+^ persistent clones correlated with the frequency of tetramer^+^ naive CD4^+^ prevaccination T cells. We conclude that the ability of RZV to recruit naive CD4^+^ T cells into the response may contribute to the durability of its effect. The abundance, breadth, and frequency of public clonotypes may further add to its protective effect.

## Graphical abstract

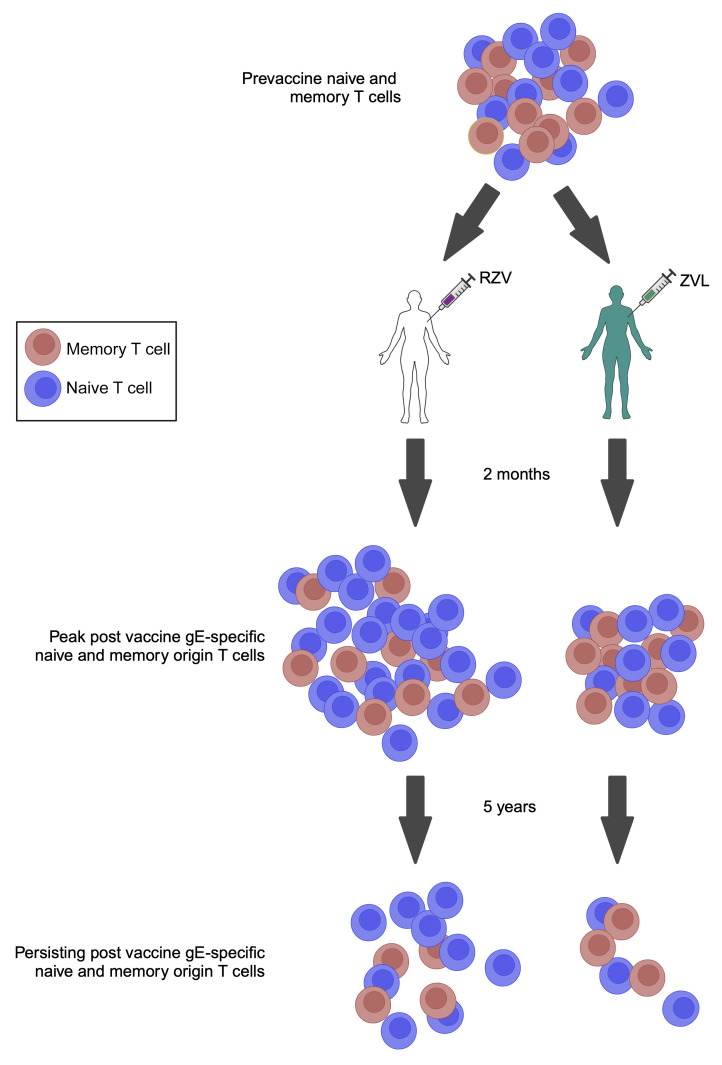

## Introduction

Herpes zoster (HZ) is a severe disease caused by reactivation of varicella-zoster virus (VZV) that is not optimally controlled by the immune system. Protection against HZ is primarily mediated by T cells (1–3). Thus, HZ primarily affects people with decreased cell-mediated immunity (CMI), such as those with congenital and acquired immunodeficiencies, including AIDS, iatrogenic immunosuppression, and older adults affected by immune senescence (1–4). Vaccination is the preferred mode of protection against many infections, including HZ, but people at the highest risk of developing the disease tend to have poor responses to vaccines. This paradigm is well exemplified by the zoster vaccine live (ZVL), which confers 70% protection against HZ in adults 50 to 69 years of age, 64% at 60 or more years of age, 41% at 70 or more years of age, and no protection at 80 or more years of age (5–7). Moreover, protection conferred by ZVL wanes significantly 3 to 5 years after vaccination and disappears within 10 years, whereas the recombinant glycoprotein E (gE) zoster vaccine (RZV) achieved a 3-year efficacy of greater than 90% and a 10-year efficacy of greater than 70% against HZ in healthy adults 50 or more years of age, including those 80 or older (8–10).

The importance of understanding the mechanisms underlying the high efficacy and durability of RZV cannot be overstated. RZV is one of the few vaccines to show similar immunogenicity in adults from 50 to more than 70 years of age. Further underscoring the uniqueness of RZV, the immunogenicity of RZV in older adults has much greater durability than many other vaccines, including mRNA vaccines (11, 12). Understanding the mechanisms underlying the strong and durable responses to RZV has wide implications for designing efficacious vaccines for older adults.

RZV contains a single VZV glycoprotein, gE, administered as two 50-μg doses (separated by 2–6 months) together with a potent adjuvant, AS01_B_ (13). In contrast, ZVL is a live, replication-competent vaccine administered as a single dose of 19,400 infectious units (at expiry) of vOKA-attenuated VZV (7). ZVL exposes the host to all VZV immunogens, including gE, which is the most abundant glycoprotein in VZV. Although each dose of ZVL contains only approximately 5.25 μg of gE (Hannah Nam, GC Biopharma; personal communication), the total amount of gE presented to the host immune system is amplified through viral replication. In our previous investigations of the immunogenicity of the HZ vaccines, we found that RZV recipients maintained higher levels of gE- and VZV-specific IL-2–producing CD4^+^ T cells for at least 5 years compared with prevaccination levels (14, 15). In contrast, ZVL recipients lost gE- and VZV-specific vaccine-induced responses within 2 years or less after immunization (14, 15). One factor that may have contributed to the superior immunogenicity of RZV is the co-administration of gE with ASO1_B_ (16). Another important observation from our studies and prelicensure clinical trials is that very few vaccinees had detectable gE-specific CMI before immunization, suggesting that the response to RZV was a primary response by many vaccine recipients (15–17). Moreover, the kinetics of the immune response to the initial RZV regimen is characteristic of primary immunization, with a modest CMI response to the first dose and a much larger response to the second dose administered 60 days later (15–17). Notably, the re-administration of RZV 10 years after the initial immunization series generated typical anamnestic responses, underscoring that the initial vaccination series served as a primary immunization (18). In contrast to RZV, the CMI responses to the single dose of ZVL is characteristic of an anamnestic response, and a second dose of ZVL administered 60 days later did not increase VZV-specific CMI compared to the first dose (19). Collectively, these observations suggested that by virtue of generating a primary response, RZV was more likely than ZVL to recruit naive CD4^+^ T cells in the memory response to vaccination. We hypothesize that memory cells derived from the naive pool may persist longer than responses derived from memory cells, which may explain the durable immunity in RZV recipients. To test this hypothesis, we compared the number, origin, diversity, and persistence of circulating gE-specific CD4^+^ T cell clonotypes selected by RZV and ZVL using prevaccine samples as a reference for preexisting memory cells.

## Results

### Demographic characteristics of the study population.

This study used cryopreserved peripheral blood mononuclear cells (PBMCs) from 5 ZVL and 16 RZV recipients who participated in a study comparing the immunogenicity of the 2 HZ vaccines (ClinicalTrials.gov NCT02114333). All participants were VZV seropositive before vaccination and immunocompetent. Participants with detectable T cell proliferation after vaccination in response to ex vivo stimulation with a pool of peptides spanning the gE glycoprotein were selected for the current study among 35 RZV and 15 ZVL vaccinees with available proliferation data. They had a mean age of 59.95 (SD 9.1) years, included 12 females, and were all White non-Hispanics (Supplemental Table 1; supplemental material available online with this article; https://doi.org/10.1172/JCI172634DS1). The demographic characteristics of RZV and ZVL recipients did not differ.

### gE-specific CD4^+^ T cell clonotypes are more abundant and diverse in RZV than ZVL recipients.

To assess the CD4^+^ T cell clonotypic response to RZV and ZVL, PBMCs obtained from 5 ZVL and 10 RZV recipients 1 month (peak response) and 5 years (persistent response) after the last dose of vaccine (single dose of ZVL and second dose of RZV) were labeled with a nontoxic protein-reactive dye and expanded ex vivo in the presence of gE-overlapping peptides (gE pp) before sorting dye-diluted proliferating CD4^+^ T cells (gating strategy shown in Supplemental Figure 1A). The median (upper; lower quartiles) frequencies of proliferating CD4^+^ T cells generated by gE pp stimulation in RZV recipients at peak and persistent response were 19% (12%; 37%) and 6.9% (5.1%; 20%), respectively (Supplemental Figure 1B). Corresponding frequencies of CD4^+^ T cells in ZVL recipients were 5.2% (3.2%; 10%) and 2.5% (1.4%; 3.5%), respectively (Supplemental Figure 1B). At both time points, significantly more CD4^+^ T cells proliferated in RZV than in ZVL recipients (*P* ≤ 0.01; Supplemental Figure 1B) and significantly more cells proliferated at peak than during persistent responses.

**Figure 1:**
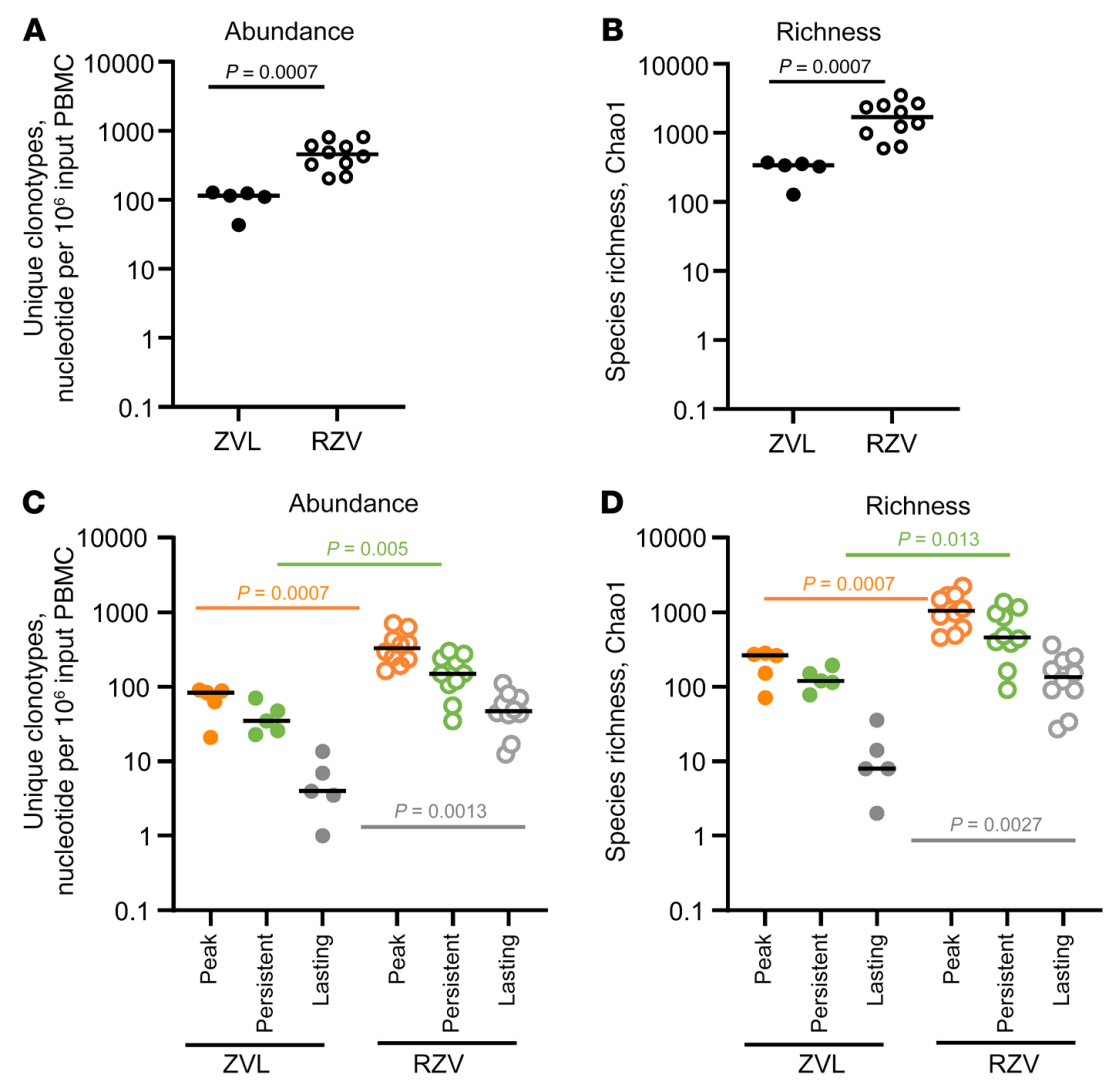
gE-reactive CD4^+^ T cell clonotypes selected by ZVL or RZV administration. Data were derived from 5 ZVL and 10 RZV recipients. CD4^+^ T cells expanded in culture for 5 days in the presence of gE peptide pools (gating strategy in Supplemental Figure 1) were submitted for *TRB* sequencing. (**A**) Combined number of unique gE-reactive CD4^+^ T cell clonotypes, normalized by number of stimulated PBMCs, identified in individual ZVL or RZV participants 30 days after the single dose of ZVL or 30 days after the second dose of RZV (peak response) and/or 5 years after vaccination (persistent response). (**B**) Diversity of the clonotypes in **A** using the Chao1 analytical approach, which weights minor populations. (**C**) Distribution of the normalized number of unique gE-reactive clonotypes present at peak, persistent, or at both time points (lasting). (**D**) Diversity of the clonotypes in **C**. The horizontal lines indicate medians in each vaccine group. *P* values, calculated with Wilcoxon’s rank-sum tests, are shown on each graph.

FACS-purified proliferating CD4^+^ T cells were submitted for quantitative T cell receptor (TCR) β chain (*TRB*) sequencing of the hypervariable region. The *TRB* analyses of peak (1 month after the last doses of vaccine) and persistent responses (5 years after the last dose of vaccine) revealed both unique and shared clonotypes between the 2 time points. Counting all CD4^+^ gE-specific clonotypes identified at peak and/or persistent response, RZV generated a significantly higher number of unique clonotypes per input PBMCs (1 × 10^6^) than did ZVL (*P* = 0.0007; Figure 1A) and had a significantly higher Chao1 diversity index, a diversity richness metric that more accurately weights minor populations such as rare TCR clonotypes (*P* = 0.0007; Figure 1B) (20). The comparison of the number and diversity of the gE-specific CD4^+^ T cell clonotypes between the 2 vaccines in each category, including peak, persistent, and lasting responses (clonotypes present both at peak and after 5 years), showed significant differences in all categories (Figure 1, C and D). The number of participants was too small to draw conclusions about the relationship with sex or age.

### RZV recruits higher proportions of naive CD4^+^ T cells into the immune response than ZVL.

**Figure 2:**
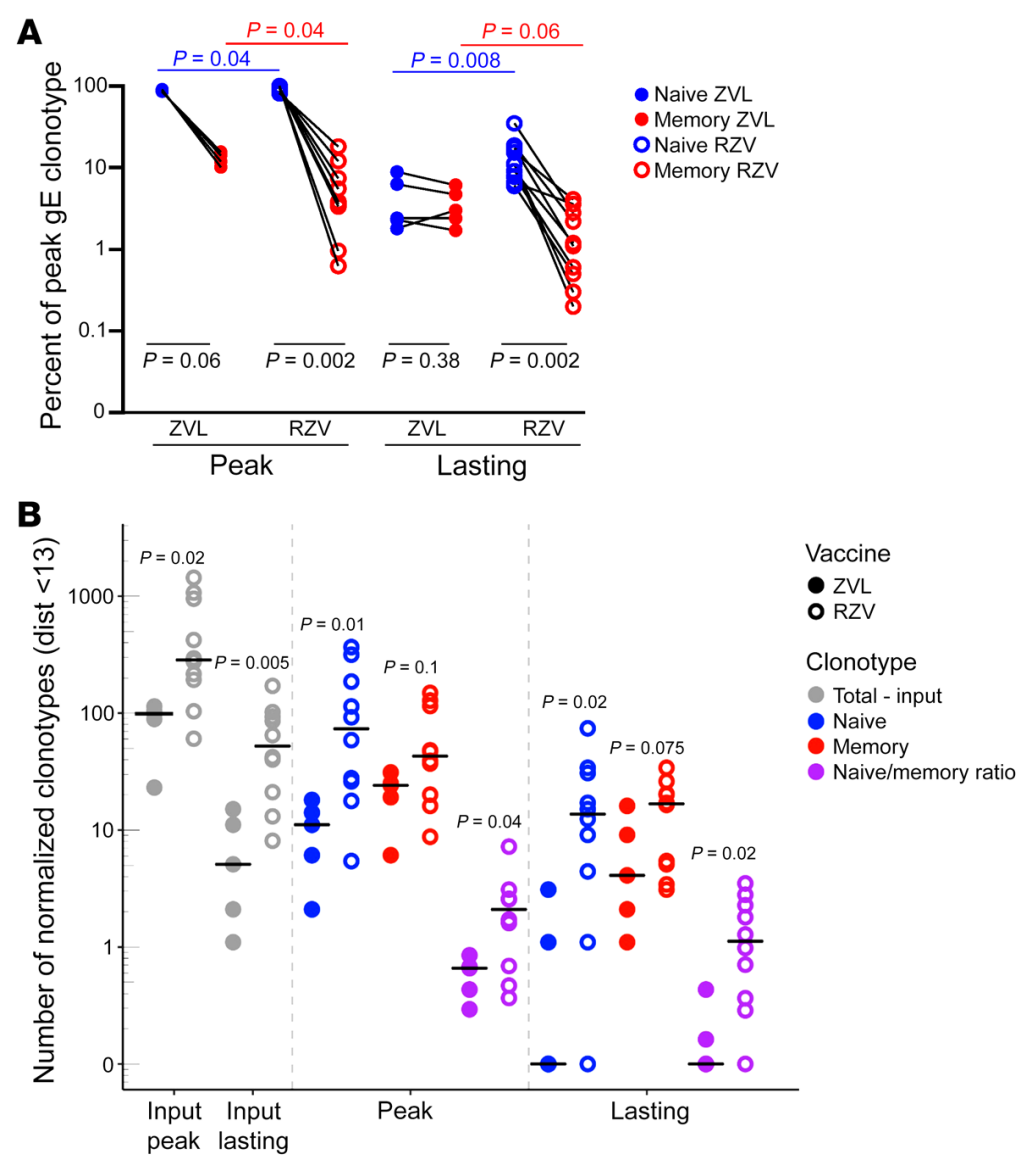
RZV expands gE-reactive CD4^+^ clonotypes mostly from naive and ZVL from memory CD4^+^ T cells. Data were derived from 10 RZV and 5 ZVL recipients. gE-reactive *TRB* identified at peak response (30 days after the single dose of ZVL or 30 days after the second dose of RZV) were matched to sorted naive and memory CD4^+^ T cells obtained before vaccination (Supplemental Figure 2). Memory indicates clonotypes exclusively matched to memory cells and naive includes all other clonotypes as described in the text. (**A**) Proportion of naive- and memory-matched peak and lasting clonotypes out of total peak clonotypes. Open circles represent results of individual RZV participants and closed circles of ZVL recipients with lines connecting paired results; *P* values for intergroup comparisons were calculated with Wilcoxon’s rank-sum test and for intragroup comparisons by Wilcoxon’s signed-rank pair test. (**B**) *TRB* clonotypes detected in sorted proliferated cells at peak only or both peak and 5-year (lasting) time points are enumerated as input and normalized to unique clonotypes per 1 × 10^6^ stimulated PBMCs. These sequences were used to query for related *TRB* sequences using the TCRdist algorithm in search sets consisting of sorted memory and naive CD4^+^ T cell populations from prior to vaccination. The normalized number of clonotypes detected per 1 × 10^6^ PBMCs with extended matching to TCRs detected at either peak or both time points (lasting) is indicated. Circles represent the normalized number per 1 × 10^6^ input PBMCs of peak and lasting clonotypes detected with extended matching to have closely related *TRB* sequences in either memory or naive bulk sequencing repertoires and the ratio of naive to memory identification. *P* values calculated with Wilcoxon’s rank-sum test.

**Figure 3:**
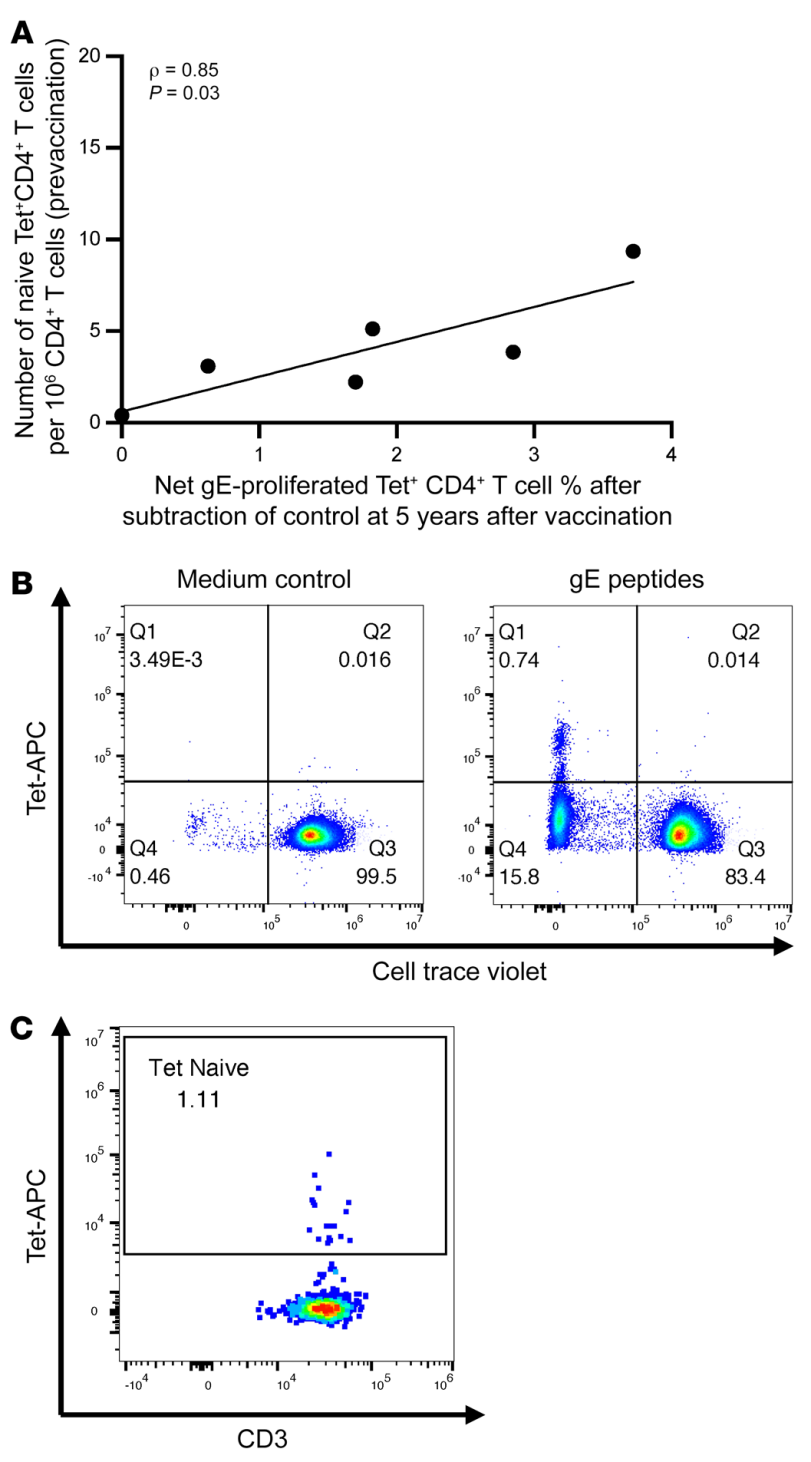
The frequency of lasting CD4^+^ T cell clones correlates with the frequency of their precursors in naive, prevaccination CD4^+^ T cells. PBMCs obtained at peak response and 5 years after RZV were labeled with CellTrace Violet and stimulated with gE peptides or medium for 10 days in the presence of rhIL-2 in the last 5 days of culture. On day 10, PBMCs were stained with anti-CD3, -CD4s, and -CD8 mAbs, and the proportion of peptide–MHC class II tetramer^+^ (Tet^+^) CD4^+^ T cells was enumerated using the gating strategy shown in Supplemental Figure 4A. For 6 participants with robust proliferated Tet^+^CD4^+^ cells at peak response and variable levels at 5 years after RZV, Tet^+^CD4^+^ T cells were enumerated in naive and memory CD4^+^ T cells obtained before vaccination using the gating strategy shown in Supplemental Figure 4B. (**A**) Shows a significant correlation between the number of CD4^+^ naive Tet^+^ T cells per 1 × 10^6^ CD4^+^ T cells depicted on the *y* axis and their corresponding proportions of Tet^+^CD4^+^ proliferated cells at 5 years in the presence of gE peptides after subtraction of medium control depicted on the *x* axis. The coefficient of correlation and *P* value shown on the graph were calculated with Spearman’s correlation test. (**B**) Shows a typical representation of Tet^+^CD4^+^ proliferated cells 5 years after vaccination in the presence of medium and gE pp, as indicated on the graph (full gating tree in Supplemental Figure 4A). (**C**) Shows a typical representation of directly ex vivo–stained Tet^+^CD4^+^ naive cells obtained before vaccination (full gating tree in Supplemental Figure 4B).

We investigated the origin of gE-specific unique clonotypes at peak response and of lasting clonotypes by matching their *TRB* sequences to those of bulk memory and naive CD4^+^ T cells isolated before vaccination (representative gating strategy shown in Supplemental Figure 2). gE-reactive CD4^+^ T cell clonotypes expanded by vaccination that were not detected before vaccination in either FACS-purified memory or naive T cells were grouped for this analysis with the naive cells. This grouping assumes that very low frequency clonotypes unseen in either the sorted and assayed memory or naive populations were more likely to be naive than memory, as memory clonotypes were likely to already have been expanded by previous encounters with the antigen. A very low number of clonotypes present both in naive and memory CD4^+^ T cell pools before vaccination were also grouped with the naive T cells. Using exact *TRB* matching criteria at the nucleotide level, the proportion of peak response gE-specific *TRB* that matched exclusively to the memory data set was 3-fold higher in ZVL recipients (median 11.9%, IQR 10.2%–14.9%) than RZV recipients (median 3.7%, IQR 2.8%–8.6%, *P* = 0.04; Figure 2A). Conversely, RZV had a significantly higher number of matches in naive cells (96% [IQR 91%–97%] versus 88% [IQR 85%–90%] in ZVL, *P* = 0.04; Figure 2A). The analysis of lasting clonotypes showed similar results (Figure 2A). ZVL recipients did not show appreciable differences between memory and naive CD4^+^ *TRB* matches among peak or lasting clonotypes (*P* = 0.06 and 0.375, respectively; Figure 2A). In contrast, RZV recipients had significantly more matches in naive than memory CD4^+^ *TRB*, both among peak and lasting clonotypes (*P* = 0.002 for both subsets; Figure 3A). A sensitivity analysis excluding clonotypes present both in memory and naive CD4^+^ T cells from the naive/non-memory pool generated similar results (Supplemental Figure 3A).

It was recently recognized that peptide-HLA complexes can bind sequence-similar TCRs, with conservative amino acid differences in hypervariable CDR3 regions (21). This allows a related swarm of T cells to participate in the immune response against each immunogenic epitope. To capture the full potential CD4^+^ T cell responses after RZV or ZVL, we used a computational sequence similarity algorithm (TCRdist) (22, 23) that identified closely related *TRB* sequences in either memory or naive prevaccination *TRB* repertoires. Using a conservative definition of amino acid similarity (up to 4 chemically similar amino acid changes in the CDR3 region or a single chemically dissimilar amino acid change or deletion) (22–24) and normalized to the input number of cells, we found that RZV recipients had significantly more clonotypes in their predicted gE-reactive swarms than ZVL recipients. This was true for sequence-related *TRB* identified by TCRdist for both peak (median 285 per RZV recipient versus 99 per ZVL recipient, *P* = 0.02; Figure 2B) and lasting (53.3 versus 5, *P* = 0.005; Figure 2B) CD4^+^ clonotypes. The extended clonotypes at peak response matched to naive CD4^+^ T cells in higher proportions in RZV than ZVL recipients (median 75.3 per person versus 11, *P* = 0.01), but in similar proportions to memory T cells (median 43 per person versus 24, *P* = 0.1). Respective ratios of naive to memory gE-specific clonotypes were 2.04 versus 0.56 (*P* = 0.04; Figure 2B). The analysis of lasting clonotypes showed similar differences to the peak response, with a significantly higher number of clonotypes derived from the naive pool and significantly higher ratios of naive to memory matched clonotypes in RZV than ZVL recipients (Figure 2B). There were no significant differences in the numbers of memory-matched lasting clonotypes between the 2 vaccines (Figure 2B). A sensitivity analysis excluding clonotypes present both in memory and naive CD4^+^ T cells generally confirmed the results (Supplemental Figure 3B). The number of participants was too small to draw conclusions about the relationship with sex or age.

To further investigate the relationship between the persistence of gE-specific CD4^+^ T cell clones and their origin in naive or memory T cell pools, we used gE-specific peptide–MHC class II tetramers (Tets). RZV recipients with class II MHC types matching the available gE Tets were screened for the presence of Tet^+^CD4^+^ T cells using PBMCs from peak and from 5 years expanded for 10 days in the presence of gE pp or unstimulated controls (Figure 3B and Supplemental Figure 4A). We selected 6 RZV recipients with greater than 1% Tet^+^CD4^+^ T cells in peak gE-stimulated cell lines after subtraction of background, which ensured that Tet^+^CD4^+^ T cells were expanded by the vaccine. Among the 6 RZV recipients, 4 had greater than 1% Tet^+^ gE-proliferated CD4^+^ T cells after subtraction of background at 5 years and 2 had less than 1% gE-proliferated Tet^+^CD4^+^ T cells at 5 years, which ensured a range of lasting responses. In these recipients, we measured the proportion of Tet^+^ cells among direct ex vivo naive CD4^+^ T cells obtained before vaccination (Figure 3C and Supplemental Figure 4B). We found a significant positive correlation (ρ = 0.85; *P* = 0.03) between the frequencies of Tet^+^ naive CD4^+^ T cells and Tet^+^ lasting CD4^+^ T clones (Figure 3A). In contrast, the correlation of Tet^+^ memory CD4^+^ T cells with Tet^+^ proliferated CD4^+^ T cells at 5 years was not significant (*P* = 0.11).

### RZV generates higher abundance of gE-specific public and related clonotypes than ZVL.

**Figure 4:**
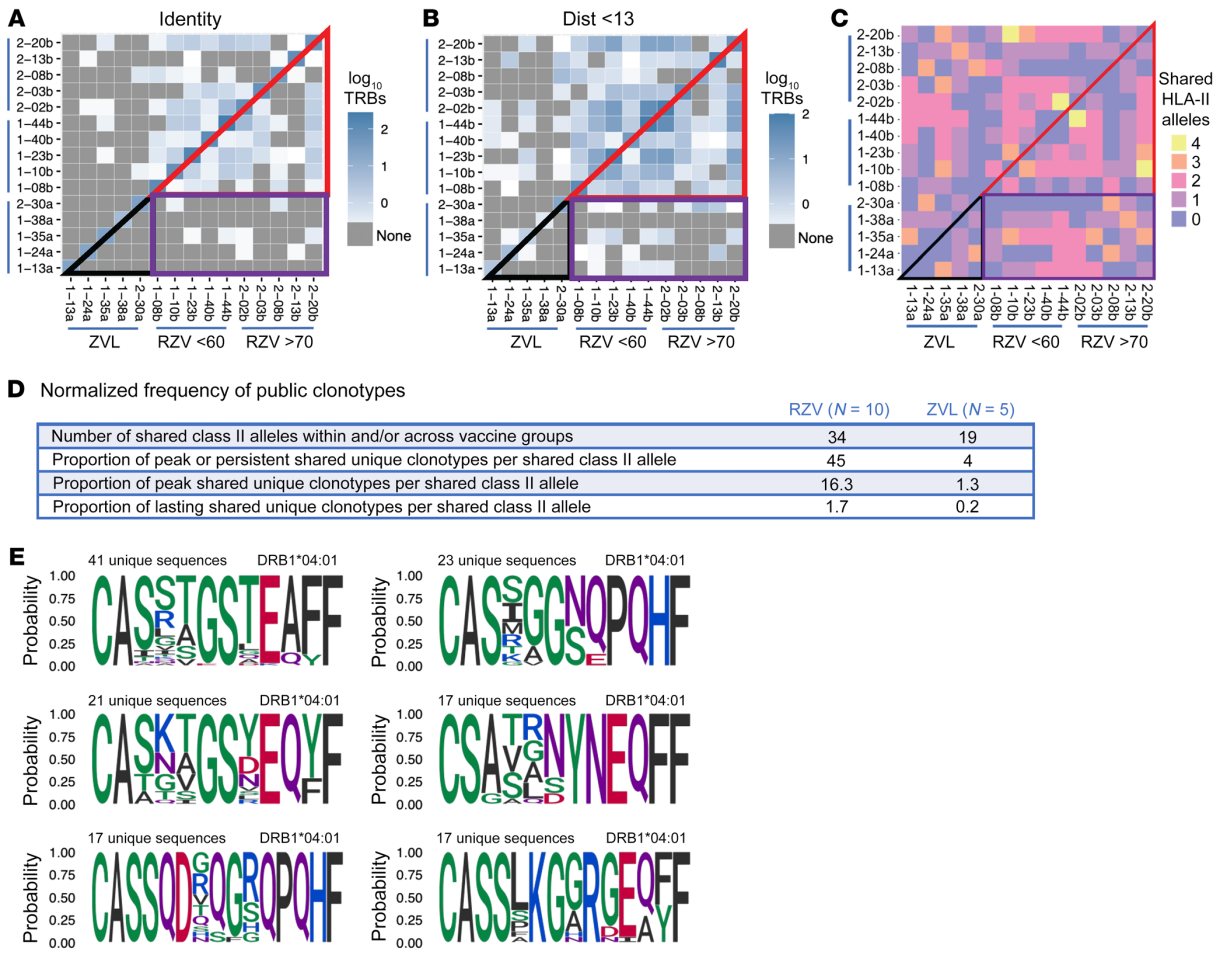
RZV expands more public clonotypes than ZVL. Data were derived from 5 ZVL recipients, including 4 at age <60 years and 1 at >70 years, and 10 RZV recipients, including 5 each at <60 and >70 years. (**A**) The heatmap shows the number of gE-reactive clonotypes with exact matches shared by RZV or ZVL recipients (legend on the right of the panel). (**B**) Number of shared clonotypes identified by computational sequence similarity algorithm (TCRdist3). (**C**) Number of MHC class II alleles shared by each participant with other participants in the ZVL or RZV group. In **A**–**C**, the lower left black triangle demarcates clonotype or allele exclusively shared among ZVL participants; the upper right red triangle exclusively shared among RZV participants; and purple rectangle shared among RZV and ZVL participants. The visual comparison of the color distribution in **A** and **B** shows much higher number of shared clonotypes among RZV participants (red triangles) than among ZVL participants (black triangles). In contrast, visual comparison of the color distribution within the red and black triangles in **C** shows similar patterns, indicating similarity in the frequency of shared HLA class II alleles across RZV and ZVL participants. (**D**) Summary of shared MHC class II alleles and proportion of peak and/or persistent, persistent only, and lasting public clonotypes identified by TCRdist normalized by the number of shared alleles. The results show a higher number of normalized public clonotypes in RZV compared with ZVL recipients in all conditions. (**E**) Amino acid sequence logo plots of 6 of the most common HLA-restricted gE-reactive CD4^+^ T cell clusters shared among multiple participants.

A public TCR clonotype is an identical V-CDR3 amino acid sequence that is shared between 2 individuals. Public clonotypes have been associated with higher avidity, immune dominance, and better control of viral replication than private clonotypes (25–28). We enumerated public clonotypes among all gE-specific clonotypes identified in ZVL and RZV recipients after vaccination. Only a few peak and/or persistent public clonotypes were observed in ZVL recipients using exact matching as a criterion (Figure 4A). Liberalizing the analytical criteria to membership in a TCR sequence–related cluster (with a conservative *TRB* sequence similarity distance <13), we found 77 expanded matches at peak and/or persistent response in ZVL recipients (Figure 4B). In contrast, RZV generated more public or related clonotypes than ZVL for exact sequence match criteria (Figure 4A) and a total of 1537 using expanded criteria (Figure 4B). Restricting the expanded analysis of public clonotypes at peak responses, ZVL had 27 while RZV had 689 unique clonotypes in shared public clusters. Corresponding numbers for lasting clonotypes were 4 and 66, respectively (Supplemental Figure 5). In contrast, the average number of shared MHC class II alleles among DP, DQ, and DR in ZVL and RZV recipients was similar (Figure 4C), with an average of 3.6 shared alleles per participant in ZVL recipients and 3.4 in RZV recipients (Figure 4D). The discrepancy between the number of shared alleles and the number of shared gE-reactive clonotypes resulted in approximately 10-fold higher numbers of putative gE-specific public clonotypes per shared MHC class II allele in RZV compared with ZVL participants at any time point (Figure 4D), demonstrating a higher propensity of RZV to select and expand public clonotypes compared with ZVL. Illustrative amino acid sequence logo plots of 6 of the most common HLA-restricted gE-reactive CD4^+^ T cell clusters found in multiple participants are presented in Figure 4E.

## Discussion

We found that the durability of the CD4^+^ T cell responses generated by RZV correlated with the ability of the vaccine to recruit naive CD4^+^ T cells into the immune response. This conclusion is supported by the following observations: (a) the frequency of lasting gE-specific CD4^+^ T cell clones correlated with the frequency of precursors among naive CD4^+^ T cells prior to vaccination, and (b) RZV preferentially recruited CD4^+^ clonotypes from the naive CD4^+^ T cell pools. These clonotypes were more likely to persist for 5 or more years after immunization than clonotypes derived from memory pools. In contrast, ZVL engaged similar proportions of clonotypes from the memory and naive pools, which was associated with an overall lower durability of the response. In a previous study, Qi et al., examining the global VZV-specific T cell response generated by ZVL, found that many of the naive CD4^+^ T cells recruited by the vaccine did not persist beyond the first few months after vaccination (29). We also noted a significant decrease in the number of gE-specific clonotypes from peak to persistent response selected both from the memory or naive pools by RZV or ZVL, but in the case of RZV, we observed that significantly higher numbers of lasting clonotypes were derived from naive than from memory T cells. The apparent important role of naive CD4^+^ T cells in the persistence of responses to vaccines, coupled with the decrease in the number of naive CD4^+^ T cells in older adults (30) may contribute to the difficulty of effectively immunizing adults 60 years of age or older. Our findings are particularly relevant to the development of new recombinant protein, vectored, and mRNA vaccines, which allow for precise antigen selection.

The discovery that TCRs that vary by a few amino acids can bind to the same MHC-presented peptide greatly expanded our understanding of the breadth of the immune responses against infectious agents (21). Here, using the TCRdist algorithm, which applies a biochemically aware matrix to give a numerical “distance” to amino acid substitutions, insertions, or deletions in comparison to a defined set of reference sequences, we were able to expand our analysis of potentially VZV gE-reactive *TRB* sequences within the precursor naive and memory TCR repertoires. We only examined *TRB*; future studies using single-cell paired sequencing could inspect TCR repertoires at the dual chain level for swarms of related TCRs both within and between individuals with greater certainty. Our analysis found that RZV compared with ZVL was associated with a greater proportion of putative gE-reactive CD4^+^ T cell clonotypes that persisted for 5 or more years after immunization. The majority of these clonotypes originated from the naive CD4^+^ T cell pools. Together with the Tet and exact-match analyses, these findings support a critical contribution made by naive CD4^+^ T cells to the persistence of the CD4^+^ T cell responses to RZV.

Another aspect of the immune response that differentiated RZV from ZVL was the selection and expansion of public clonotypes. After their initial descriptions (31), public clonotypes have been identified against multiple antigens, including HIV and herpesviruses, such as cytomegalovirus and Epstein-Barr virus, and herpes simplex, a virus closely related to VZV (32). The probability of the same clonotype being selected by multiple individuals by chance is so low that alternative explanations were sought for their existence. Recent work has shown that public clonotypes have high affinity for their cognate epitopes. In addition, public epitopes may be able to tolerate a small number of mutations in the epitope being recognized, or in their CDR3 sequences, without losing the ability to recognize cognate antigen (25–28). Public clonotypes may also be favored because of a high probability of generation (Pgen) arising from low genomic distances between V, D, J, and C genes in the TCR locus and a low number of insertions/deletions required to create the final TCR nucleotide sequences (21). Collectively, these properties suggest that public clonotypes may confer superior protection against infectious agents, which is consistent with the observation that people with HIV who control viral replication without antiretrovirals, also known as elite controllers, have higher frequencies of public clonotypes than noncontrollers (28). We propose that the ability to elicit public clonotypes may contribute to the high efficacy of RZV and may also be a desirable characteristic for other vaccines.

The ability of RZV to elicit public clonotypes is likely to be a function of the magnitude and breadth of the T cell response to the gE antigen in the vaccine, both of which were much greater than the response of ZVL recipients. Notably, ZVL recipients mount immune responses against multiple VZV gene products in addition to gE. Although we do not know the overall frequency of public clonotypes elicited by ZVL, it is conceivable that narrowing the immune response to a single glycoprotein may result in selection of more public clonotypes than diffusing the immune response across multiple antigens. This hypothesis deserves to be further studied. The amount of gE in RZV and the co-administration of a potent adjuvant may also contribute to the selection of public clonotypes.

Preexisting immune responses to pathogens related to VZV could influence the antigenicity of zoster vaccines. Among the 8 human herpesviruses (HHV), HHV-1 and HHV-2, better known as HSV-1 and HSV-2, are the most similar to VZV with regards to gene organization and protein sequence. Indeed, there are several known examples of T cell cross-reactivity between specific peptide epitopes in VZV and HSV-1/HSV-2, including 1 well-defined epitope in the gE homologs in all 3 viral species that is recognized by CD4^+^ T cells (33). It is also remarkable that infection with either HSV-1 or HSV-2, or both, appears to be protective against the development of shingles in individuals in the placebo arm of a randomized control trial of ZVL (34), indicating that cross-reactive acquired immunity may have functional importance when considered at the whole-virus level. However, the sequence of VZV gE is quite divergent overall from that of HSV-1 or HSV-2 gE, with only limited regions of high homology or identity. We did not define the HSV infection status of individuals in this report, but low overall gE protein similarity makes it unlikely that HSV-driven cross-reactive memory responses influenced our results.

Limitations of this study include the relatively small number of samples and cells tested, which allowed us to detect only differences with large effect sizes. Results generated with small sample sizes may be subject to sampling errors and to bias by a few outliers. We strove to minimize the effect of outlier observations by using nonparametric statistical tests. Another limitation was the use of single-chain TCR sequencing. TCRs that recognize the same peptide-MHC complex may sometimes be weighted toward either *TRB* or *TRA* chains of the TCR heterodimer (35). Future studies could investigate responses to zoster vaccines at the single-chain, paired *TRB/TRA* level.

In conclusion, RZV is distinguished from ZVL by the ability to recruit long lasting clonotypes from the naive CD4^+^ T cell pool, by the magnitude and breadth of the immune response, and by selection of public clonotypes.

## Methods

### Design of the parent study.

The study enrolled 160 participants in good health except for treated chronic illnesses typical of the age of the vaccinees. All had prior varicella or resided in the United States at least 30 years and subsequent antibody testing by gp ELISA confirmed the presence of VZV-specific antibodies in all participants (36); none had prior HZ. Exclusions from the study were immune suppression and recent blood products or other vaccines. The participants were divided between 2 age groups of 50–59 years old (*n* = 46) or 70–85 years old (*n* = 115). The older group included 70 people who received ZVL 5 or more years before enrollment and 44 who did not. Participants in each group were randomized at enrollment to receive 1 dose of ZVL followed by placebo 60 days later or RZV in 2 doses separated by 60 days (Supplemental Figure 6). Of 160 enrollees, 159 were vaccinated. The current study used blood samples collected before vaccination, at peak response (30 days after the last dose of vaccine corresponding to study day 30 for ZVL and 90 for RZV recipients), and 5 years after immunization from participants who did not receive prior ZVL.

### Isolation of gE-specific CD4^+^ T cells.

Postvaccine PBMCs from peak response and year 5 time points were thawed, stained with CellTrace Violet (CTV) (BioLegend), and cultured for 5 days in the presence of gE 15mer peptides overlapping by 11 amino acids (MilliporeSigma custom order based on the Genbank sequence Q9J3M8; Supplemental Table 2) at 2.5 μg/mL final concentration of each peptide in growth medium consisting of RPMI 1640 (Mediatech) with L-glutamine (Gemini BioProducts), 10% human AB serum (Gemini BioProducts), 2% HEPES (Mediatech), and 1% penicillin-streptomycin (Gemini BioProducts). Unstimulated controls were incubated in growth medium only. At the end of the incubation, PBMCs were washed and stained with Zombie Yellow Viability Stain (BioLegend). PBMCs were then stained with anti-CD3–Alexa Fluor 700 (clone UCHT1, BD Biosciences), anti-CD4–PerCPCy5.5 (clone A161A1, BioLegend), and anti-CD8–PE-CF594 (clone RPA-T8, BD Biosciences). The gE-specific CD4^+^ T cells were identified by the CD4^+^CTV^dim^ populations and then isolated by FACS using a Beckman Coulter Astrios EQ. The median number of cells sorted was 18,400 (range 1000–119,000) at peak and 3500 (range 770–63,000) at 5 years. Supplemental Figure 1A shows an example of the gating strategy.

### Memory/naive subset sorting.

Prevaccine PBMC samples were stained with a cocktail containing anti-CD3–ECD (clone UCHT1, Thermo Fisher Scientific), anti-CD8–FITC (clone 3B5, Life Technologies), anti-CD4–BV510 (clone A161A1, BioLegend), anti-CD45RA-APC (clone HI100, BioLegend), anti-CD95–Pacific Blue (clone DX2, BioLegend), anti-CCR7-PE (clone G043H7, BioLegend), and 7-AAD (Thermo Fisher Scientific). Naive (CCR7^+^CD45RA^+^CD95^–^) and memory (not CCR7^+^CD45RA^+^) live CD3^+^CD8^–^CD4^+^ T cells were isolated by FACS using a BD FACSAria 2 (University of Washington Cell Analysis Facility). Supplemental Figure 2 shows an example of the gating strategy.

### TRB sequencing.

Sorted cells were pelleted and frozen at –80°C. DNA for CTV-diluted cell populations was extracted using a DNeasy Blood/Tissue Kit (Qiagen). DNA extraction for naive and memory CD4^+^ T cell populations was performed by Adaptive Biotechnologies. Survey (gE-specific) and deep (memory, naive) level *TRB* sequencing was performed at Adaptive Biotechnologies (ImmunoSEQ Assay TCRBv4b). Data were exported from the Adaptive Immunoseq platform and analyzed in R v4.0.1 (https://www.r-project.org/) and Python v3.7 (https://www.python.org/). Out-of-frame or noncoding sequences and those without valid V or J gene assignments were excluded. For exact clonotype tracking, clonotypes were considered to be present in multiple samples if they had the same CDR3 nucleotide sequence and *TRBV* and *TRBJ* genes. Sequences were considered likely to represent gE-reactive if they were present (in the gE samples) at 2 or more copies; single *TRB* sequences were excluded.

### Sequence similarity analysis.

Pairwise dissimilarity was computed between the gE-reactive *TRB* clonotypes and the memory and naive bulk repertoires using TCRdist3 v0.2.2 (22, 23). A conservative dissimilarity level of less than 13 distance units was used (which would allow a maximum of 4 amino acid differences within the CDR3 region) to identify additional potentially gE-reactive swarms of *TRB* sequences within the bulk repertoires. An HLA feasibility analysis (35) was used to assign likely HLA restriction to specific public clusters; results were confirmed by HLA-shared-allele clustering wherein only persons with specific shared HLA alleles were iteratively included in the clustering algorithm. Likely HLA alleles were assigned to a cluster only if there was a single possible HLA restriction and if the cluster was observed in more than 3 persons. TCR motif visualization was performed using ggseqlogo and ggplot2 in R v4.0.1. Logo plots demonstrate position-specific frequency of each amino acid within a *TRB* cluster.

### Cultured PBMC Tet staining.

Postvaccine PBMCs from peak response and year 5 time points were thawed, stained with CTV, and cultured in the presence of gE peptide pools (2.5 μg/mL) or mock stimulation for 10 days. On day five, 5 IU/mL rhIL-2 (R&D Systems) was added to mock and gE wells. At the end of the incubation, PBMCs were washed and stained with Zombie Yellow Viability Stain. Tet staining was performed for 1 hour at 37°C using VZV gE-specific APC-conjugated HLA-DRB1*04:01 EIEPGVLKVLRTEKQYLGVY, HLA-DRB1*15:01 PQPRGAEFHMWNYHSHVFSV, HLA-DRB1*07:01 RIYGVRYTETWSFLPSLTCT, or HLA-DRB1*11:04 EIEPGVLKVLRTEKQYLGVY (Benaroya Research Institute Tetramer Core Laboratory). PBMCs were then stained with anti-CD3–APC-H7 (clone SK7, BD Biosciences), anti-CD4–PerCPCy5.5 (clone RPA-T4, BD Biosciences), and anti-CD8–PE-CF594 (clone RPA-T8, BD Biosciences). Cells were acquired using the NovoCyte Quanteon flow cytometer (Agilent Technologies), and the Tet^+^ cells were identified in the live, single-cell, CD3^+^CD8^–^CD4^+^CTV^lo^ population using FlowJo analysis software (BD Biosciences) (Supplemental Figure 4A).

### Ex vivo PBMC Tet staining.

Prevaccine PBMCs were thawed and stained with Zombie Yellow Viability Stain. Anti–human CD32 blocker (STEMCELL Technologies) was added at a dilution of 1:100. Tet staining was performed as above. APC-positive cells were selected using magnetic bead enrichment as per the manufacturer’s instructions (STEMCELL Technologies). The bound magnetic particles were removed from the selected cells following enrichment. The Tet-enriched cells were stained with anti-CD3–APC-H7 (clone SK7, BD Biosciences), anti-CD4–PerCPCy5.5 (clone RPA-T4, BD Biosciences), anti-CD8–PE-CF594 (clone RPA-T8, BD Biosciences), anti-CD95-BV421 (clone DX2, BioLegend), anti-CCR7-FITC (clone G043H7, BioLegend), and anti-CD45RA–PE-Cy7 (clone HI100, BioLegend). The cells were acquired using the NovoCyte Quanteon flow cytometer and analyzed using FlowJo analysis software (Supplemental Figure 4B).

### Statistics.

In all analyses, the number of clonotypes was normalized to input PBMCs. Wilcoxon’s signed-rank tests were used to compare paired (within person) distribution, and Wilcoxon’s rank-sum tests were used to compare distributions between vaccine groups. Spearman’s test was used for correlation analysis. *P* values were not corrected for multiple comparisons. Statistical testing was performed in R v4.0.1 and Prism v9.0 (GraphPad).

### Study approval.

The study (NCT02114333) was approved by the Colorado Multiple Institutional Review Board. All participants provided signed informed consent.

### Data availability.

All *TRB* sequences are available at Zenodo (https://doi.org/10.5281/zenodo.7872753). The study-specific code used for repertoire processing is available on GitHub (link: https://gist.github.com/esford3/0b49ea55adacb96ee29412573a178ab7). The analysis was otherwise performed with publicly available packages (TCRdist3, ggseqlogo). Supporting Data Values for graphs are included in the supplemental material. Additional data are available by request from the corresponding author.

## Author contributions

KJL designed and performed *TRB* sequencing experiments and participated in data analysis and manuscript preparation. ESF performed TCRdist analysis and participated in data analysis and manuscript preparation. MJJ performed flow cytometry assays and participated in data analysis and manuscript preparation. MJL led the parent study and participated in manuscript preparation. DMK participated in study design, data analysis, and manuscript preparation. AW conceptualized and designed the study, participated in data analysis, and led the manuscript preparation. The order of authorship for KJL and ESF in the author list reflects the chronological order of their participation in the study.

## Supplementary Material

Supplemental data

Supporting data values

## Version 1 10/03/2023

In-Press Preview

## Version 2 12/01/2023

Electronic publication
