# Supplementary material for: Recruitment of naive CD4+ T cells by the recombinant zoster vaccine correlates with persistent immunity
Source: J Clin Invest. 2023 Dec 1;133(23):e172634. doi: 10.1172/JCI172634 (PMC10688978; doi:10.1172/JCI172634)
Supplement: Supplemental data [file jci-133-172634-s069.pdf]

Table S1. Demographic Characteristics of the Study Population

Characteristic	RZV (N=16)	ZVL (N=5)	Overall (N=21)
Mean years of age (s.d.)	60.3 (9.7)	58.6 (7.3)	59.95 (9.1)
Number of females	8	4	12
White Non-Hispanics	16	5	21

Supplemental Table 2. Amino acid sequences of the peptides in the VZV gE pool

Peptide Name	N-Terminal Mod.	Sequence	C-Terminal Mod.
Genbank Q9J3M8 1	[H]	MGTVNKPVVGVLMGF	[OH]
Genbank Q9J3M8 2	[H]	NKPVVGVLMGFGIIT	[OH]
Genbank Q9J3M8 3	[H]	VGVLMGFGIITGTLR	[OH]
Genbank Q9J3M8 4	[H]	MGFGIITGTLRITNP	[OH]
Genbank Q9J3M8 5	[H]	IITGTLRITNPVRAS	[OH]
Genbank Q9J3M8 6	[H]	TLRITNPVRASVLRY	[OH]
Genbank Q9J3M8 7	[H]	TNPVRASVLRYDDFH	[OH]
Genbank Q9J3M8 8	[H]	RASVLRYDDFHIDED	[OH]
Genbank Q9J3M8 9	[H]	LRYDDFHIDEDKLDT	[OH]
Genbank Q9J3M8 10	[H]	DFHIDEDKLDTNSVY	[OH]
Genbank Q9J3M8 11	[H]	DEDKLDTNSVYEPYY	[OH]
Genbank Q9J3M8 12	[H]	LDTNSVYEPYYHSDH	[OH]
Genbank Q9J3M8 13	[H]	SVYEPYYHSDHAESS	[OH]
Genbank Q9J3M8 14	[H]	PYYHSDHAESSWVNR	[OH]
Genbank Q9J3M8 15	[H]	SDHAESSWVNRGESS	[OH]
Genbank Q9J3M8 16	[H]	ESSWVNRGESSRKAY	[OH]
Genbank Q9J3M8 17	[H]	VNRGESSRKAYDHNS	[OH]
Genbank Q9J3M8 18	[H]	ESSRKAYDHNSPYIW	[OH]
Genbank Q9J3M8 19	[H]	KAYDHNSPYIWPRND	[OH]
Genbank Q9J3M8 20	[H]	HNSPYIWPRNDYDGF	[OH]
Genbank Q9J3M8 21	[H]	YIWPRNDYDGFLENA	[OH]
Genbank Q9J3M8 22	[H]	RNDYDGFLENAHEHH	[OH]
Genbank Q9J3M8 23	[H]	DGFLENAHEHHGVYN	[OH]
Genbank Q9J3M8 24	[H]	ENAHEHHGVYNQGRG	[OH]
Genbank Q9J3M8 25	[H]	EHHGVYNQGRGIDSG	[OH]
Genbank Q9J3M8 26	[H]	VYNQGRGIDSGERLM	[OH]
Genbank Q9J3M8 27	[H]	GRGIDSGERLMQPTQ	[OH]
Genbank Q9J3M8 28	[H]	DSGERLMQPTQMSAQ	[OH]
Genbank Q9J3M8 29	[H]	RLMQPTQMSAQEDLG	[OH]
Genbank Q9J3M8 30	[H]	PTQMSAQEDLGDDTG	[OH]
Genbank Q9J3M8 31	[H]	SAQEDLGDDTGIHVI	[OH]
Genbank Q9J3M8 32	[H]	DLGDDTGIHVIPTLN	[OH]
Genbank Q9J3M8 33	[H]	DTGIHVIPTLNGDDR	[OH]
Genbank Q9J3M8 34	[H]	HVIPTLNGDDRHKIV	[OH]
Genbank Q9J3M8 35	[H]	TLNGDDRHKIVNVDQ	[OH]
Genbank Q9J3M8 36	[H]	DDRHKIVNVDQRQYG	[OH]
Genbank Q9J3M8 37	[H]	KIVNVDQRQYGDVFK	[OH]
Genbank Q9J3M8 38	[H]	VDQRQYGDVFKGDLN	[OH]
Genbank Q9J3M8 39	[H]	QYGDVFKGDLNPKPQ	[OH]
Genbank Q9J3M8 40	[H]	VFKGDLNPKPQGQRL	[OH]
Genbank Q9J3M8 41	[H]	DLNPKPQGQRLIEVS	[OH]
Genbank Q9J3M8 42	[H]	KPQGQRLIEVSVEEN	[OH]
Genbank Q9J3M8 43	[H]	QRLIEVSVEENHPFT	[OH]
Genbank Q9J3M8 44	[H]	EVSVEENHPFTLRAP	[OH]
Genbank Q9J3M8 45	[H]	EENHPFTLRAPIQRI	[OH]
Genbank Q9J3M8 46	[H]	PFTLRAPIQRIYGVR	[OH]
Genbank Q9J3M8 47	[H]	RAPIQRIYGVRYTET	[OH]
Genbank Q9J3M8 48	[H]	QRIYGVRYTETWSFL	[OH]
Genbank Q9J3M8 49	[H]	GVRYTETWSFLPSLT	[OH]
Genbank Q9J3M8 50	[H]	TETWSFLPSLTCTGD	[OH]
Genbank Q9J3M8 51	[H]	SFLPSLTCTGDAAPA	[OH]
Genbank Q9J3M8 52	[H]	SLTCTGDAAPAIQHI	[OH]
Genbank Q9J3M8 53	[H]	TGDAAPAIQHICLKH	[OH]
Genbank Q9J3M8 54	[H]	APAIQHICLKHTTCF	[OH]

**Genbank Q9J3M8 55**	[H]	QHICLKHTTCFQDVV	[OH]
**Genbank Q9J3M8 56**	[H]	LKHTTCFQDVVVDVD	[OH]
**Genbank Q9J3M8 57**	[H]	TCFQDVVVDVDCAEN	[OH]
**Genbank Q9J3M8 58**	[H]	DVVVDVDCAENTKED	[OH]
**Genbank Q9J3M8 59**	[H]	DVDCAENTKEDQLAE	[OH]
**Genbank Q9J3M8 60**	[H]	AENTKEDQLAEISYR	[OH]
**Genbank Q9J3M8 61**	[H]	KEDQLAEISYRFQGK	[OH]
**Genbank Q9J3M8 62**	[H]	LAEISYRFQGKKEAD	[OH]
**Genbank Q9J3M8 63**	[H]	SYRFQGKKEADQPWI	[OH]
**Genbank Q9J3M8 64**	[H]	QGKKEADQPWIVVNT	[OH]
**Genbank Q9J3M8 65**	[H]	EADQPWIVVNTSTLF	[OH]
**Genbank Q9J3M8 66**	[H]	PWIVVNTSTLFDELE	[OH]
**Genbank Q9J3M8 67**	[H]	VNTSTLFDELELDPP	[OH]
**Genbank Q9J3M8 68**	[H]	TLFDELELDPPEIEP	[OH]
**Genbank Q9J3M8 69**	[H]	ELELDPPEIEPGVLK	[OH]
**Genbank Q9J3M8 70**	[H]	DPPEIEPGVLKVLRT	[OH]
**Genbank Q9J3M8 71**	[H]	IEPGVLKVLRTEKQY	[OH]
**Genbank Q9J3M8 72**	[H]	VLKVLRTEKQYLGVY	[OH]
**Genbank Q9J3M8 73**	[H]	LRTEKQYLGVYIWNM	[OH]
**Genbank Q9J3M8 74**	[H]	KQYLGVYIWNMRGSD	[OH]
**Genbank Q9J3M8 75**	[H]	GVYIWNMRGSDGTST	[OH]
**Genbank Q9J3M8 76**	[H]	WNMRGSDGTSTYATF	[OH]
**Genbank Q9J3M8 77**	[H]	GSDGTSTYATFLVTW	[OH]
**Genbank Q9J3M8 78**	[H]	TSTYATFLVTWKGDE	[OH]
**Genbank Q9J3M8 79**	[H]	ATFLVTWKGDEKTRN	[OH]
**Genbank Q9J3M8 80**	[H]	VTWKGDEKTRNPTPA	[OH]
**Genbank Q9J3M8 81**	[H]	GDEKTRNPTPAVTPQ	[OH]
**Genbank Q9J3M8 82**	[H]	TRNPTPAVTPQPRGA	[OH]
**Genbank Q9J3M8 83**	[H]	TPAVTPQPRGAEFHM	[OH]
**Genbank Q9J3M8 84**	[H]	TPQPRGAEFHMWNYH	[OH]
**Genbank Q9J3M8 85**	[H]	RGAEFHMWNYHSHVF	[OH]
**Genbank Q9J3M8 86**	[H]	FHMWNYHSHVFSVGD	[OH]
**Genbank Q9J3M8 87**	[H]	NYHSHVFSVGDTFSL	[OH]
**Genbank Q9J3M8 88**	[H]	HVFSVGDTFSLAMHL	[OH]
**Genbank Q9J3M8 89**	[H]	VGDTFSLAMHLQYKI	[OH]
**Genbank Q9J3M8 90**	[H]	FSLAMHLQYKIHEAP	[OH]
**Genbank Q9J3M8 91**	[H]	MHLQYKIHEAPFDLL	[OH]
**Genbank Q9J3M8 92**	[H]	YKIHEAPFDLLLEWL	[OH]
**Genbank Q9J3M8 93**	[H]	EAPFDLLLEWLYVPI	[OH]
**Genbank Q9J3M8 94**	[H]	DLLLEWLYVPIDPTC	[OH]
**Genbank Q9J3M8 95**	[H]	EWLYVPIDPTCQPMR	[OH]
**Genbank Q9J3M8 96**	[H]	VPIDPTCQPMRLYST	[OH]
**Genbank Q9J3M8 97**	[H]	PTCQPMRLYSTCLYH	[OH]
**Genbank Q9J3M8 98**	[H]	PMRLYSTCLYHPNAP	[OH]
**Genbank Q9J3M8 99**	[H]	YSTCLYHPNAPQCLS	[OH]
**Genbank Q9J3M8 100**	[H]	LYHPNAPQCLSHMNS	[OH]
**Genbank Q9J3M8 101**	[H]	NAPQCLSHMNSGCTF	[OH]
**Genbank Q9J3M8 102**	[H]	CLSHMNSGCTFTSPH	[OH]
**Genbank Q9J3M8 103**	[H]	MNSGCTFTSPHLAQR	[OH]
**Genbank Q9J3M8 104**	[H]	CTFTSPHLAQRVAST	[OH]
**Genbank Q9J3M8 105**	[H]	SPHLAQRVASTVYQN	[OH]
**Genbank Q9J3M8 106**	[H]	AQRVASTVYQNCEHA	[OH]
**Genbank Q9J3M8 107**	[H]	ASTVYQNCEHADNYT	[OH]
**Genbank Q9J3M8 108**	[H]	YQNCEHADNYTAYCL	[OH]
**Genbank Q9J3M8 109**	[H]	EHADNYTAYCLGISH	[OH]
**Genbank Q9J3M8 110**	[H]	NYTAYCLGISHMEPS	[OH]
**Genbank Q9J3M8 111**	[H]	YCLGISHMEPSFGLI	[OH]
**Genbank Q9J3M8 112**	[H]	ISHMEPSFGLILHDG	[OH]

**Genbank Q9J3M8 113**	[H]	EPSFGLILHDGGTTL	[OH]
**Genbank Q9J3M8 114**	[H]	GLILHDGGTTLKFVD	[OH]
**Genbank Q9J3M8 115**	[H]	HDGGTTLKFVDTPES	[OH]
**Genbank Q9J3M8 116**	[H]	TTLKFVDTPESLSGL	[OH]
**Genbank Q9J3M8 117**	[H]	FVDTPESLSGLYVFV	[OH]
**Genbank Q9J3M8 118**	[H]	PESLSGLYVFVVYFN	[OH]
**Genbank Q9J3M8 119**	[H]	SGLYVFVVYFNGHVE	[OH]
**Genbank Q9J3M8 120**	[H]	VFVVYFNGHVEAVAY	[OH]
**Genbank Q9J3M8 121**	[H]	YFNGHVEAVAYTVVS	[OH]
**Genbank Q9J3M8 122**	[H]	HVEAVAYTVVSTVDH	[OH]
**Genbank Q9J3M8 123**	[H]	VAYTVVSTVDHFVNA	[OH]
**Genbank Q9J3M8 124**	[H]	VVSTVDHFVNAIEER	[OH]
**Genbank Q9J3M8 125**	[H]	VDHFVNAIEERGFPP	[OH]
**Genbank Q9J3M8 126**	[H]	VNAIEERGFPPTAGQ	[OH]
**Genbank Q9J3M8 127**	[H]	EERGFPPTAGQPPAT	[OH]
**Genbank Q9J3M8 128**	[H]	FPPTAGQPPATTKPK	[OH]
**Genbank Q9J3M8 129**	[H]	AGQPPATTKPKEITP	[OH]
**Genbank Q9J3M8 130**	[H]	PATTKPKEITPVNPG	[OH]
**Genbank Q9J3M8 131**	[H]	KPKEITPVNPGTSPL	[OH]
**Genbank Q9J3M8 132**	[H]	ITPVNPGTSPLLRYA	[OH]
**Genbank Q9J3M8 133**	[H]	NPGTSPLLRYAAWTG	[OH]
**Genbank Q9J3M8 134**	[H]	SPLLRYAAWTGGLAA	[OH]
**Genbank Q9J3M8 135**	[H]	RYAAWTGGLAAVVLL	[OH]
**Genbank Q9J3M8 136**	[H]	WTGGLAAVVLLCLVI	[OH]
**Genbank Q9J3M8 137**	[H]	LAAVVLLCLVIFLIC	[OH]
**Genbank Q9J3M8 138**	[H]	VLLCLVIFLICTAKR	[OH]
**Genbank Q9J3M8 139**	[H]	LVIFLICTAKRMRVK	[OH]
**Genbank Q9J3M8 140**	[H]	LICTAKRMRVKAYRV	[OH]
**Genbank Q9J3M8 141**	[H]	AKRMRVKAYRVDKSP	[OH]
**Genbank Q9J3M8 142**	[H]	RVKAYRVDKSPYNQS	[OH]
**Genbank Q9J3M8 143**	[H]	YRVDKSPYNQSMYYA	[OH]
**Genbank Q9J3M8 144**	[H]	KSPYNQSMYYAGLPV	[OH]
**Genbank Q9J3M8 145**	[H]	NQSMYYAGLPVDDFE	[OH]
**Genbank Q9J3M8 146**	[H]	YYAGLPVDDFEDSES	[OH]
**Genbank Q9J3M8 147**	[H]	LPVDDFEDSESTDTE	[OH]
**Genbank Q9J3M8 148**	[H]	DFEDSESTDTEEEFG	[OH]
**Genbank Q9J3M8 149**	[H]	SESTDTEEEFGNAIG	[OH]
**Genbank Q9J3M8 150**	[H]	DTEEEFGNAIGGSHG	[OH]
**Genbank Q9J3M8 151**	[H]	EFGNAIGGSHGGSSY	[OH]
**Genbank Q9J3M8 152**	[H]	AIGGSHGGSSYTVYI	[OH]
**Genbank Q9J3M8 153**	[H]	SHGGSSYTVYIDKTR	[OH]

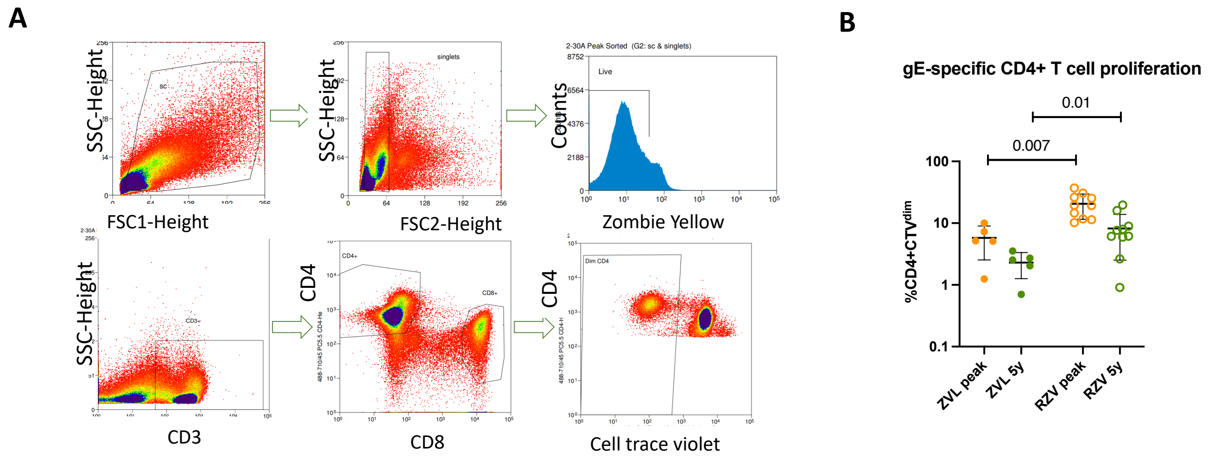

Supplemental Figure 1. gE-specific CD4+ T cell selection. PBMC obtained at peak response (30 days after the single dose of ZVL or 30 days after the 2nd dose of RZV) and 5 years after vaccination from 5 ZVL and 10 RZV recipients were thawed. Viability of the PBMC varied from 78.4% to 99.9%. Cells were labeled with Cell Trace Violet (CTV) and expanded for 5 days in the presence of gE peptide pools. **Panel A** shows the gating strategy. **Panel B** summarizes the proportion of proliferated CD4+ T cells. Circles represent number of cells sorted from each participant. Horizontal lines represent means and S.D. in each vaccine group. The p values were generated with Mann-Whitney test.

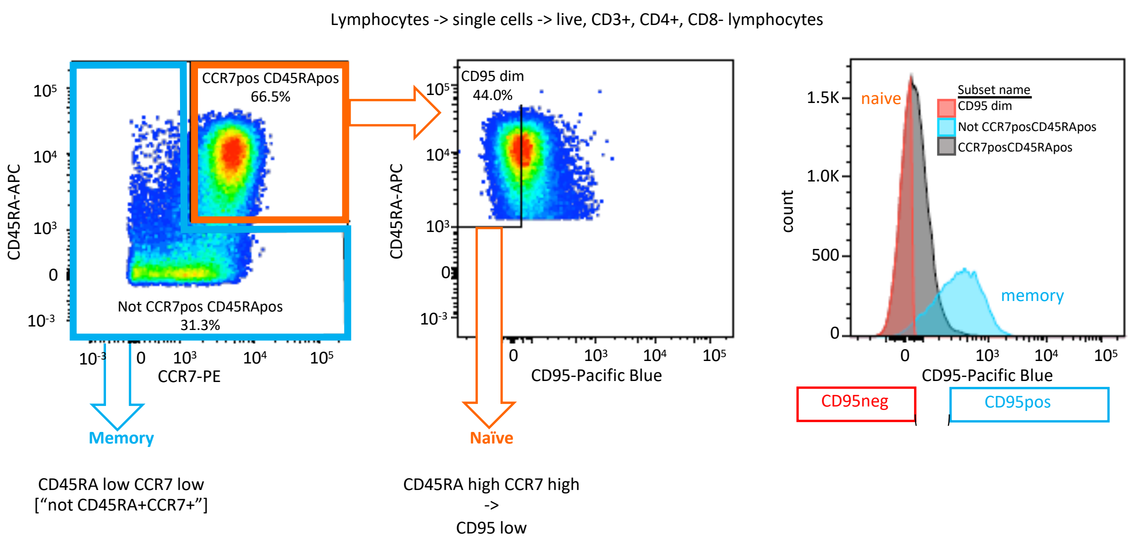

Supplemental Figure 2. Gating strategy for sort-purification of CD4+ naïve and memory T cells. The expression of CD95 was analyzed on CD4+CCR7+CD45RA+ cells (middle panel) and CD95- cells were isolated as naïve cells.

A

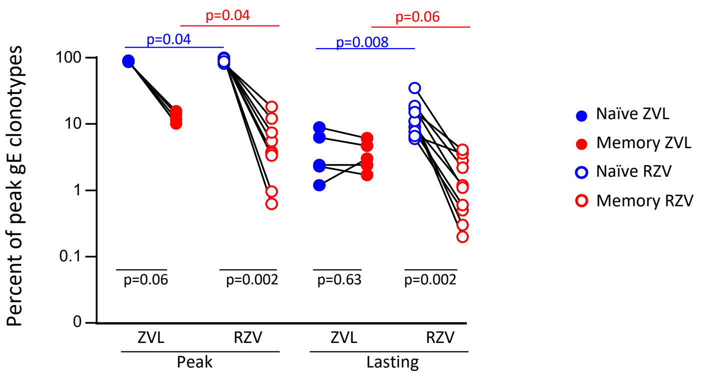

B

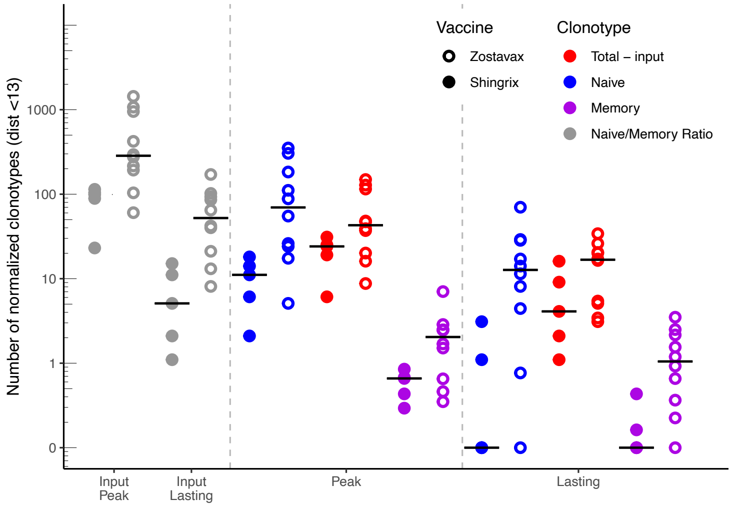

Supplemental Figure 3. RZV expands gE-reactive CD4+ clonotypes mostly from naïve and ZVL from memory CD4+ T cells (sensitivity analysis). Data were derived from 10 RZV and 5 ZVL recipients. gE-reactive *TRB* identified at peak response (30 days after the single dose of ZVL or 30 days after the 2nd dose of RZV) were matched to sorted naïve and memory CD4+ T cells obtained pre-vaccination (**Fig S2**). In this sensitivity analysis, memory indicates clonotypes exclusively matched to memory cells and naive includes all other clonotypes after the exclusion of clonotypes matched both in naïve and memory CD4+ T cells. **Panel A**: Proportion of naïve- and memory-matched peak and lasting clonotypes out of total peak clonotypes. Circles represent

results of individual participants; horizontal lines represent medians; p values for inter-group comparisons were calculated with Wilcoxon rank sum tests and for intra-group comparisons by Wilcoxon signed-rank pair test. **Panel B**: *TRB* clonotypes detected in sorted proliferated cells at peak only or both peak and 5-year (lasting) time points are enumerated as input and normalized to unique clonotypes per 10^6 stimulated PBMC. These sequences were used to query for related *TRB* sequences using the TCRdist algorithm in search sets comprised of sorted memory and naïve CD4+ T cell populations from prior to vaccination. The normalized number of clonotypes detected per 10^6 PBMC with extended matching to TCRs detected at either peak or both time points (lasting) is indicated. Circles represent the normalized number per 10^6 input PBMC of peak and lasting clonotypes detected with extended matching to have closely related *TRB* sequences in either memory or naive bulk sequencing repertoires and the ratio of naive to memory identification. P values calculated with Wilcoxon rank sum test.

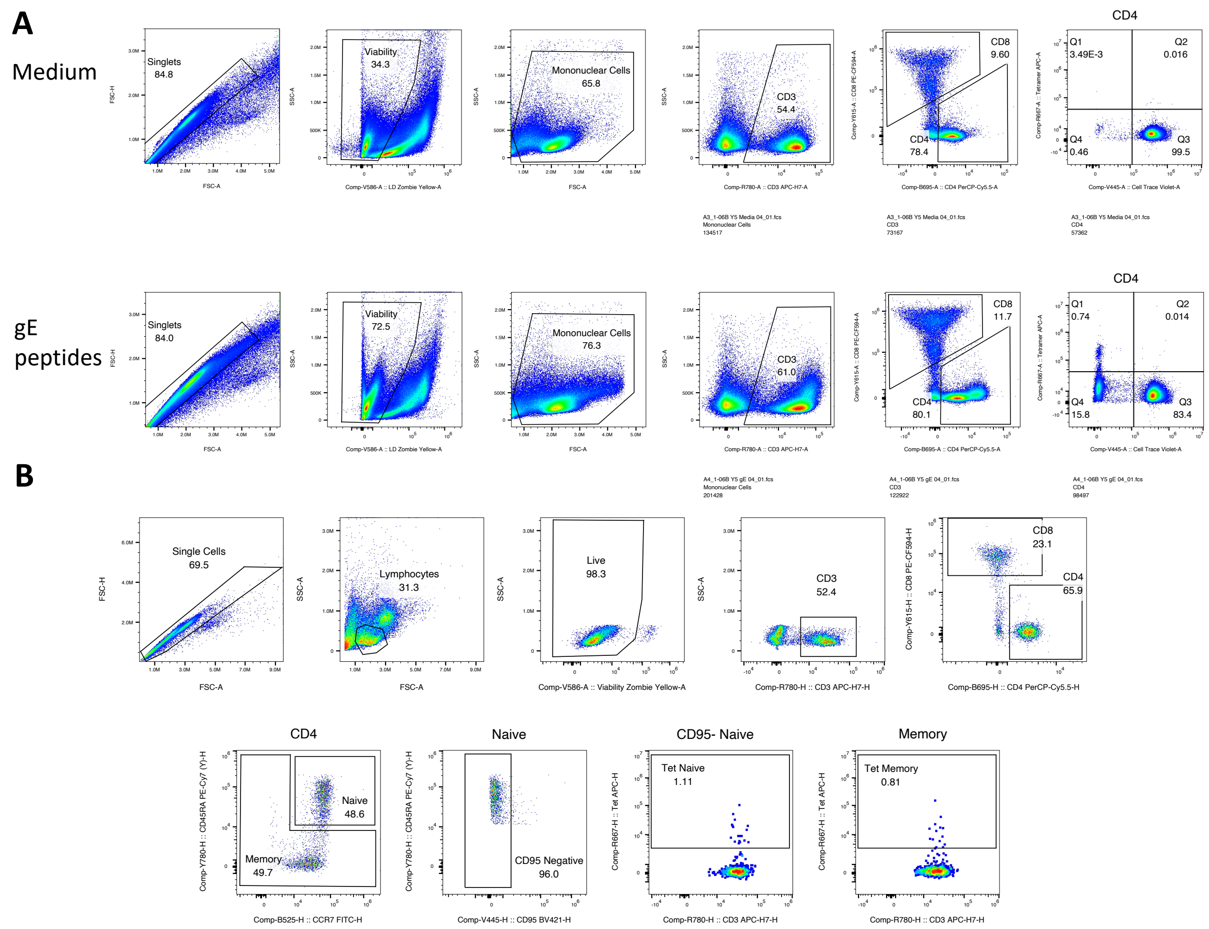

Supplemental Figure 4. Gating strategy for experiments using tetramer staining. A: Gating tree for in vitro expanded CD4+ T cells. PBMC obtained 5 years after vaccination were expanded in the presence of Medium or gE peptides, as indicated on the graph, for 10 days with addition of rhIL-2 during the last 5 days of culture. On day 10, PBMC were stained with CD3, CD4 and CD8 mAb, and with APC-conjugated p-MHC class II tetramers. The proportion of Tet+ cells in the gE-stimulated proliferated CD4+ T cells after subtraction of medium control measured clonotype persistence. **B: Gating tree for identifying Tet+ memory and naïve CD4+ T cells prevaccination.** PBMC obtained before vaccination were stained with APC-conjugated pMHC class II Tet. After enrichment on anti-APC coated magnetic beads, PBMC were stained

with mAb anti-CD3, CD4, CD8, CD45RA, CD95 and CCR7 for enumeration of Tet+ cells among naïve CD5RA+CCR7+CD95- naive or CD45RA-CD95+ memory CD4+ T cells.

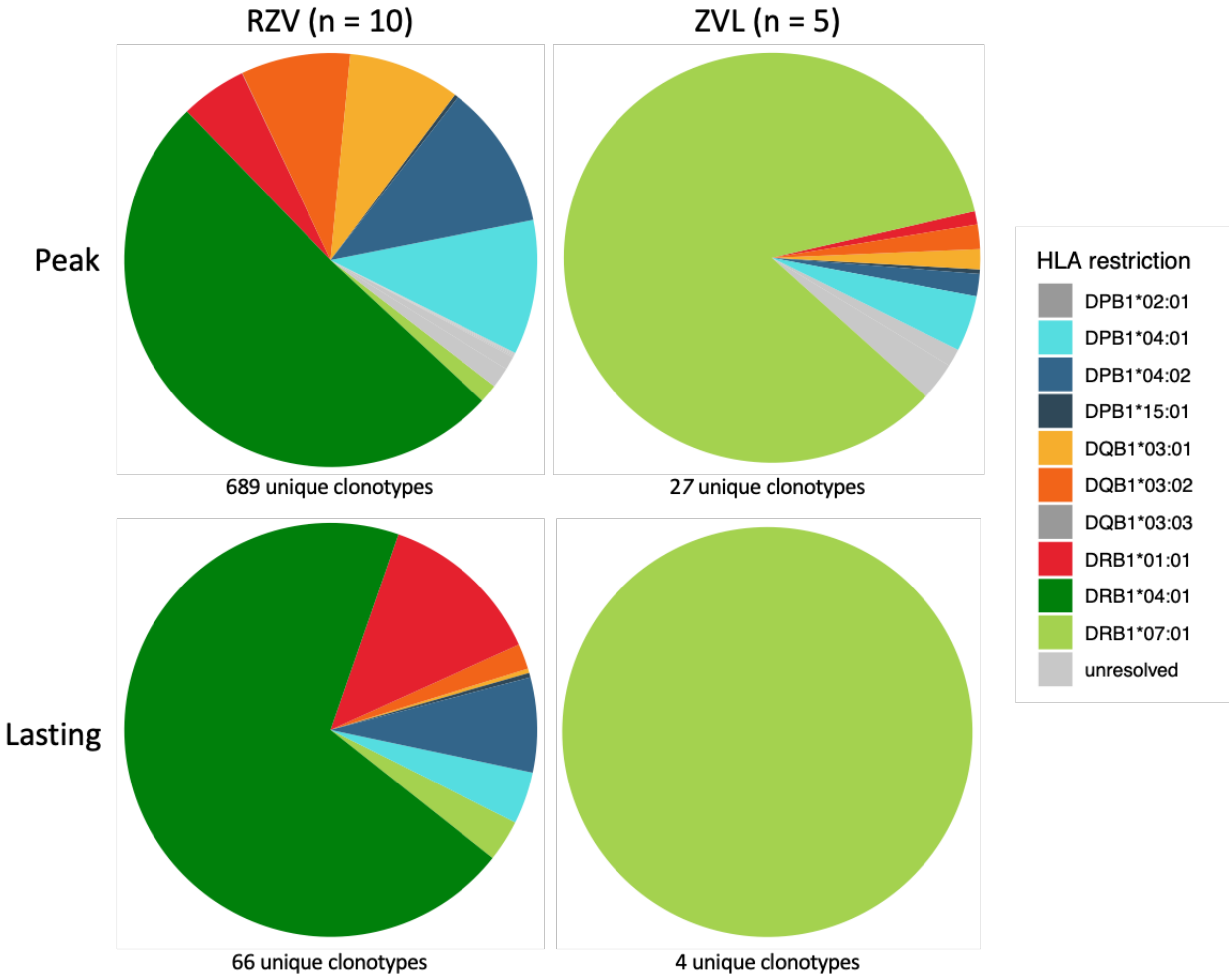

Supplemental Figure 5. Peak and lasting gE-specific public clonotypes identified by TCRdist analysis in RZV and ZVL recipients. Pie charts show the proportions of clonotypes corresponding to shared alleles. Numbers under each pie chart indicate the total number of unique clonotypes contributing to clusters. Unresolved indicates clonotypes that were not able to be linked to a single HLA allelic restriction.

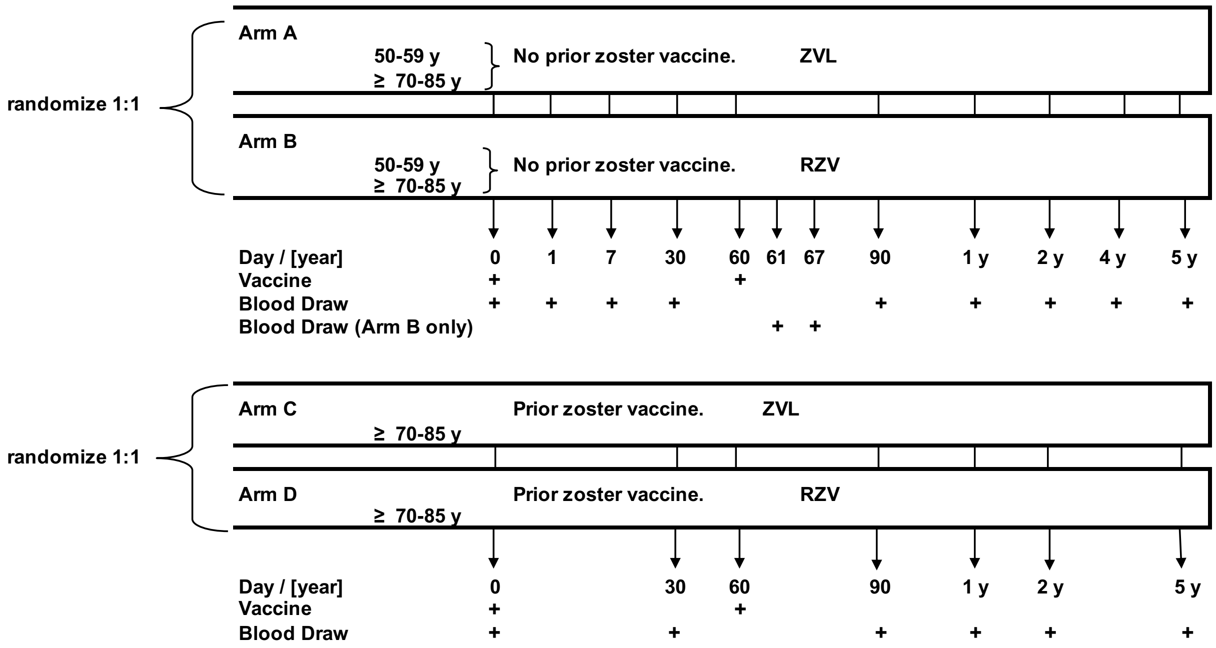

Supplemental Figure 6. Schematic representation of the parent study (NCT0211433).
